# The Ws of Parental Help-Seeking: When, Where, and for What Do Parents Seek Help for Child Mental Health

**DOI:** 10.1007/s10578-024-01683-5

**Published:** 2024-03-20

**Authors:** Vilas Sawrikar, Cheryl Van Dyke, Amy M. Smith Slep

**Affiliations:** 1 Centre for Applied Developmental Psychology, University of Edinburgh, Edinburgh, UK; 2 Department of Clinical and Health Psychology, University of Edinburgh, Edinburgh, UK; 3 Stony Brook University, Stony Brook, New York, NY USA; 4 Family Translational Research Group, New York University, New York, NY USA

**Keywords:** Childhood, Mental health, Parental help-seeking, Treatment gap

## Abstract

Parental help-seeking preferences may help explain the treatment gap in child mental health. This study examined mothers’ and fathers’ help-seeking behaviors for child mental health to further understand their individual preferences for treatment. A total of 394 mothers and fathers completed questionnaires assessing the types of help sought for mental health concerns for a target child (age 3–7 years), as well as measures representing illness profile, predisposing characteristics, and barriers/facilitators proposed to influence help-seeking. Parents often sought informal rather than professional help. Regression modelling indicated mothers’ different help-seeking behaviors were significantly associated with illness profile (marital quality, child mental health, parental education), predisposing factors (parental attributions, child age), and family income, while fathers’ different help-seeking behaviors were significantly associated with child demographics (age, gender). The results support expanding treatments into nonclinical settings and improving child mental health literacy to improve appropriate parental help-seeking for child mental health concerns.

**Supplementary Information:**

The online version contains supplementary material available at 10.1007/s10578-024-01683-5.

Early onset mental health problems in childhood are associated with higher risk for lifetime mental disorders, which incur significant costs to the child, family, and society [1, 2]. Improving access to treatment for childhood-onset problems is therefore recognized as a high priority for healthcare [3]. However, research findings indicate that relatively few children in need of help receive treatment for mental health problems [4]. Low rates of access to treatment are partially attributed to parents’ help-seeking preferences for informal help from nonprofessionals (e.g., friends, family, [5]) rather than professional help. Thus, researchers have suggested that access to treatment may be improved by changing traditional clinical service models reliant on professionals delivering treatment to also include nonclinical pathways [6]. To gain a better understanding of parents’ help-seeking preferences for child mental health problems in a community sample, the current study examined parental informal and professional help-seeking behaviors and the factors that distingusih families seeking informal versus professional help.

Research examining utilization of mental health services for child mental health problems consistently shows a treatment gap whereby there is a discrepancy between the proportion of children in need of treatment and the proportion receiving treatment [7]. Previous findings from epidemiological research indicate that approximately 80% of children under 12 years of age who need a mental health evaluation fail to receive one, and that only approximately 30% of children with diagnosable mental disorder receive treatment for that disorder [8, 9]. These research findings suggest children with mental health difficulties largely remain untreated. Researchers have attributed the treatment gap in child mental health to multiple barriers, organized around system-related (cost of mental health services, policy and legal constraints), attitudinal (stigma, mental health literacy, cultural and ethnic influences), and clinical practice (case identification, model of treatment delivery method) factors that impede families’ treatment utilization [10]. Reducing the treatment gap is therefore proposed to require the development of treatment delivery systems that can overcome these barriers [6]. This includes innovations in disseminating treatments in contexts, locations, and formats that best suit parents rather than relying on traditional treatment delivery systems [6, 7].

It is important to understand individual differences in parental help-seeking behaviors for child mental health because these indicate their preferences, all things considered, for accessing treatment. Parental help-seeking in this context refers to parents’ efforts to seek help from others to help address their children’s problems [11]. Longstanding theories propose parental help-seeking exists on a continuum, starting with seeking help from an informal sphere of family and friends as the lowest level of help-seeking, which then gives way to more authoritative forms of help (e.g., teachers, clergy) until professional help (e.g., physicians, mental health professional) is sought as the highest level of help-seeking [11–14]. Models of parental help-seeking also propose the levels of help sought for child problems are influenced by three groups of determinants: (a) *illness profile,* representing clinical and subjective need and family impact, (b) *predisposing characteristics,* representing stable factors that influences parent’s readiness to seek help, and (c) *barriers/facilitators of help-seeking* that represent social and environmental factors that impact help-seeking (see Table 1,[13, 15]). Parents are expected to seek higher levels of help in the context of more severe problems and greater family impact, as long as receiving help from authoritative figures and professionals is congruent with social-cultural norms for professional help-seeking [11].

**Table 1:** Summary of determinants hypothesized to influence parents’ decision to seek help for child mental health (e.g., [13])

Determinants	Domains	Example variables
Illness profile	Clinical and subjective assessment of need (child, parent, family)	Disability/role functioning, symptoms/behaviors, diagnoses, wellbeing, definition of behavior as a mental health concern
	Family characteristics (structural and relational)	Family size, parental education, warmth, cohesion, dis/organization, family/marital conflict - abuse/neglect, parental psychopathology, criminality
Predisposing characteristics	Demographic characteristics	Age, gender, ethnicity
	Sociocultural values/beliefs (child, parent, family)	Values/attitudes/knowledge concerning health and illness; ethnic identity; help-seeking desirability; coping strategies/efficacy; religion
Barriers/Facilitators	Community and social networks	Residential mobility, network strength, helpfulness, openness to outsiders, knowledge about and use of services, congruence of network attitudes toward use of services
	Economic factors	Income, health insurance

Despite longstanding models of parental help-seeking, there is a surprising lack of research investigating factors that determine levels of parental help-seeking for child mental health. Previous research has tended to focus on professional help-seeking and service utilization (see [16, 17] for reviews). Results from these reviews suggest that seeing professionals for help is predicted by older child age, severity, impairment, and comorbidity. Further, service utilization is greater for externalizing problems compared to internalizing problems, which may explain why help is sought more for boys than girls. Findings also indicate that service utilization increases with the degree to which the child problem interferes with parents own well-being and family functioning. Service utilization is likely when mothers report distress, or when children live in single parent, low-income, or ethnic minority household. In contrast, much less research has focused on the determinants of informal help-seeking. Results from four studies examining different levels of help-seeking have consistently shown that parents are most likely to seek informal help [5, 14, 18, 19]. Despite this, analyses examining the process of parental help-seeking in these studies do not focus on determinants of informal and professional help-seeking separately, or differences between mothers’ and fathers’ parental help-seeking behaviors despite growing evidence of inter-parental differences in preferences for treatment [20]. There is a notable lack of research examining inter-parental differences in help-seeking preferences specifically, with previous research either disregarding parent gender when reporting help-seeking behaviors or considering single parent status as a determinant of parental help-seeking [16]. For instance, Luo and colleagues [18] examined determinants of *any* parental help-seeking for socioemotional development of their 3-year-old child from variables representing predisposing factors, illness profile, and barriers/facilitators. Results indicated that general parental help-seeking was associated with ethnicity, parent’s age, and previous help-seeking, suggesting these individual characteristics may have utility in identifying families more or less likely to seek help for child problems. To that end, similar analyses for each level of informal and professional help for both mothers and fathers may help refine our understanding of help-seeking processes that can elucidate methods of matching treatment delivery to families.

The aim of the current study was to further investigate individual differences in parental help-seeking behaviors for child mental health and the individual factors that putatively influence seeking informal vs. professional help. First, we report the distribution of parental help-seeking for child mental health concern among community-based parents. No hypothesis is proposed given that the analysis was descriptive. The second aim was to examine the influence of illness profiles, predisposing characteristics, and barriers/facilitators in determining parents’ levels of help-seeking for child mental health concern. This second aim was split into two questions: (a) what the determinants of each level of parental help-seeking are compared to not seeking help from others, and (b) what the determinants of informal help-seeking are compared to professional help. It was hypothesized that higher levels of parental help-seeking would be associated with greater severity and impact in the child’s illness profile and predisposing characteristics (i.e., male child, older child, non-ethnic group status, higher religiosity, lower self-efficacy, negative parental attributions), and reporting lower help-seeking barriers (i.e., community and social networks, higher socioeconomic status).

## Method

### Study Design

The current study uses a cross-sectional observational survey design to examine the influence of illness profile, predisposing characteristics, and help-seeking barriers/facilitators on parents’ levels of help-seeking for child mental health. The data for this study is based on information collected using a battery of questionnaires.

### Participants and Recruitment

We recruited participants for a study on family conflict with the approval of the [21] University’s Institutional Review Board using a random digit dialing procedure targeting families living within a 1-h radius of the university where the study was conducted (Suffolk County, New York, United States). Specific details of family identification and contact are provided in previous studies [22, 23]. Families were eligible to participate if a heterosexual couple had been cohabiting for at least one year, were raising at least one child between the ages of 3 and 7, the child was the biological offspring of at least one parent, and they were able to complete questionnaires in English. We used these eligibility criteria because this study focused, in part, on intimate partner violence, which is affected by sex and gender. All eligible families identified through the random digit dialing survey were contacted by a senior staff member who explained the study in more detail and scheduled interested participants’ visit to the laboratory. A total of 453 heterosexual couples provided consent to participate. Details regarding sample-to-US Census comparisons are provided in previous studies [21].

We selected a subset of participants from the original study for the current study based on valid responses to the help-seeking questionnaire. We excluded families if parents indicated that the child had an intellectual disability, or physical or sensory handicap. This made it more likely that parents’ help-seeking behaviors were primarily based on child mental health concerns. The final sample consisted of *N* = 394 mothers and fathers independently reporting on a target child between the ages of 3 and 7. A summary of parents’ demographic characteristics is presented in Table 2. Families’ incomes were representative for the geographic area. Parents were in the 30–40 age range, mothers’ interquartile range: (32–38 years), fathers’ interquartile range (33–41 years)﻿. Most parents were indicated to have come from ‘white’ backgrounds, married, and have college-level education. Most fathers in the sample were employed full-time, while mothers were equally distributed across unemployed, part-time, and full-time. Most parents were biological parents of the target child. There was equal representation of male and female children.

**Table 2:** Descriptive Statistics for participant demographics

	Mothers	Fathers
Parent information		
Age (years) *M* (SD)	34.97 (4.96)	37.12 (5.87)
Family income (in thousands) *M* (SD)	81.65 (43.18)	81.65 (43.18)
Family size	4.57 (1.17)	4.57 (1.17)
Ethnicity *N* (%)		
White	322 (81.7%)	311 (78.9%)
Minority	72 (18.3%)	83 (21.1%)
Marital status *N* (%)		
Married	371 (94.2%)	371 (94.2%)
Living together	23 (5.8%)	23 (5.8%)
Employment status *N* (%)		
Unemployed	128 (32.5%)	17 (4.3%)
Part-time	149 (37.8%)	8 (2%)
Full-time	117 (29.7%)	369 (93.7%)
Education in years *M* (SD)	14.17 (2.2)	14.20 (2.32)
Relation to target child *N* (%)		
Biological parent of child	393 (99.7%)	372 (94.4%)
Step	0 (0.00%)	12 (3%)
Other	1 (.3%)	372 (94.4%)
Child information		
Age (years) *M* (SD)	5.44 (1.48)	5.44 (1.48)
Child gender *N* (%)		
Male	187 (47.5%)	187 (47.5%)
Female	207 (52.5%)	207 (52.5%)

Sample size: *N* = 394

### Procedure

Comprehensive details of study procedures can be found in [21]. In summary, eligible couples were invited to take part in the study in a laboratory located at the university. Couples were informed that the study’s aim was to learn about how families cope with conflict and why they handle problems in the ways they do. We collected data in accordance with ethical guidelines provided by the Human Research Ethics Committee. Participation in the study was voluntary, and we obtained informed consent from parents prior to participation. Couples completed a series of tasks consisting of an extensive battery of questionnaires, as well as observational and experimental tasks. Participation lasted approximately 6-h and couples could complete the protocol in one or two visits. Only responses from the battery of questionnaires were used for the current study. Couples completed study measures in separate, private rooms, and all participants completed a standard set of measures that were given in the exact same order. Responses were kept anonymous and confidential; couples were informed of this verbally and in writing during the informed consent procedure. All couples were paid $250 for their participation.

### Measures

#### Parental Concerns and Help-Seeking Behaviors

##### Parental Help-Seeking Questionnaire (PHSQ)

The PHSQ was created for this study to assess parental help-seeking behaviors for child mental health concerns. The PHSQ was adapted from the Actual Help Seeking Questionnaire (AHSQ; [24]) for assessing parental help-seeking behaviors. Parents were asked to freely report up to five concerns they remember having with the target child in the last year and then for each concern choose what type(s) of help they sought for that concern. Parents were asked to check as many of six options of help-seeking for each concern. Answer options were (a) *I sought no help (I didn’t talk to anyone, and I didn’t look for any information)*, (b) *I tried to work it out by myself by reading books, searching on the internet, or self-help methods*, (c) *I talked to friends and family about it*, (d) *I talked to my child’s teacher*, (e) *I talked to someone from the community, such as a minister or group leader*, and (f) *I talked to a professional, such as a therapist or pediatrician*. Free responses of parental concerns were coded using a protocol developed for the study (see supplementary information). Concerns were coded: (i) ‘externalizing’ for oppositional, disruptive, hyperactive, inattentive, and aggressive behaviors (e.g., “throws temper tantrums” and “is aggressive”); or (ii) ‘internalizing’ for sad and anxious behavior (e.g., “clinginess” and “seems anxious”). Responses not coded into these mental health categories were grouped into ‘other.’ Parental concerns were coded by two independent raters with high agreement (mothers, 85.94%; fathers, 88.07%). Associated help-seeking behaviors were reported as four-subscales: no help sought, help sought from family/friends; help sought from community; professional help. Responses were subsequently aggregated across the four subscales into variables indicating the highest level of parental help-seeking for concerns with externalizing or internalizing behaviors. The internal reliability for this final variable was moderate-good (α_mothers_ = 0.74; α_fathers_ = 0.61).

#### Child and Family Illness Profile

##### Child Mental Health

The child’s mental health was assessed using the externalizing and internalizing subscales of the Child Behavior Checklist (CBCL; [25]). The CBCL is a 100-item questionnaire designed to measure child psychopathology as reported by the parent. Parents rate how true each item is for their child on a 3-point scale ranging from 0 (“*Not True”*) to 2 (“*Very True or Often True”*). The CBCL is a well-validated and reliable questionnaire utilized to measure psychopathology in children [25]. Parents of children who were three years old completed the CBCL 2/3 and parents of children who were four years old or older completed the CBCL/4–18.

##### Parental Mental Health

The Beck Depression Inventory (BDI, [26]) was used to measure parental mental health. The BDI consists of 21 items assessing different cognitive, behavioral, and affective components of depression. Respondents rank each item on a 4-point scale ranging from 0 to 3. A total score was derived by summing item scores with higher scores representing more severe depressive symptomatology. The internal consistency for the Depression subscale was excellent (α_mothers_ = 0.90, α_fathers_ = 0.87).

##### Family Structural Characteristics

Structural characteristics of the family were assessed in relation to family size calculated as number of related people in the household and parental education calculated as parent’s education in years. Parental education was scored on a 6-point scale from 10th grade or less through doctoral degrees.

##### Family Relational Characteristics

The relational qualities of the family were assessed using multiple measures. First, the general functioning scale of the Family Assessment Device (FAD, [27]) was used to measure family functioning. The FAD general functional scale is a 12-item subscale of the original questionnaire measuring the family’s structural, organizational, and transactional characteristics. Participants are asked to rate how well statements of family dysfunction describe their own family on a 4-point Likert scale of agreement (1 ‘strongly agree’ to 4 ‘strongly disagree’). A total scale score of general functioning was calculated using mean level response across items, with higher scores indicating worse level of functioning. The internal consistency of the FAD general functioning scale in the current study was high (mothers, α = 0.88,fathers, α = 0.88).

Second, the Dyadic Adjustment Scale (DAS, [28]) was used to measure the quality of the couple’s relationship. The DAS is a 32-item questionnaire (DAS-32) assessing relationship satisfaction, intimacy, affective expression, and couple agreement. Participants are asked to rate their level of agreement of how well the DAS statement describe their relationship on varying ordinal response scales. A total DAS-32 score was calculated by summing responses across items, with higher scores indicating positive relationship quality. The internal consistency of the DAS-32 total scale in the current study was high (mothers, α = 0.94,fathers, α = 0.93).

#### Predisposing Characteristics

##### Demographic Characteristics

Child’s age and gender (1 = male, 2 = female) and parent’s gender and ethnic background (1 = white, 2 = ethnic) were included.

##### Religiosity

Participants rated their level of religiosity using a single item ‘how religious are you?’ on a 7-point Likert rating scale from 1 ‘Not at all’ to 7 ‘Extremely.’

##### Parental Explanations for Child Problems

Parental explanations concerning child problems were measured using the Parent Cognition Scale (PCS, [29]). The PCS is a 30-item self-report measure assessing parent’s dysfunctional attributions for their children’s problematic behavior that includes parent causal attributions (e.g., ‘I’m not structured enough with my child’) and child responsible attributions (e.g., ‘My child purposely tries to get me angry’). Participants rated how well statements describe their explanations for child behavior on a 6-point Likert scale from 0 ‘always true’ to 5 ‘never true’. The internal consistency for both the parent causal attributions (mothers, α = 0.81; fathers, α = 0.85) and child responsible attributions (mothers, α = 0.90; fathers, α = 0.88) scales was excellent.

##### Self-Efficacy Regarding Childrearing

Parental self-efficacy for childrearing was assessed using the efficacy subscale of the Parenting Sense of Competency (PSOC-efficacy, [30]). The PSOC-efficacy subscale consists of 7-items evaluating the parent’s self-efficacy in the parenting role (e.g., ‘Being a parent is manageable, and any problems are easily solved’). Parents indicate their level of agreement with each item on a 6-point Likert scale from 1 (strongly agree) to 6 (strongly disagree). A total score is calculated by summing item responses. The internal consistency for PSOC-efficacy subscale was high (mothers, α = 0.86; fathers, α = 0.87).

#### Barrier/Facilitators

##### Perceptions of Social Support

Perceptions of social support were assessed using the Interpersonal Support Evaluation List (ISEL, [31]). The ISEL consists of 40 items that are rated true or false in relation to ways that others affect their responses to events. The total number of items rated as true was used to indicate level of perceived social support.

##### Income

Family income was assessed as the total of the fathers’ and mothers’ self-reported personal income.

### Analytic Strategy

Data analyses for this study was conducted using SPSS version 25. Preliminary analysis examined the highest level of help sought (no help, self-help, family/friends, teachers, community leader, professional help) for concerns with externalizing and internalizing behavior. Subsequently, descriptive statistics (mean, standard deviation, frequency) was used to compare parents’ levels of help-seeking according to *illness profile* (child mental health [CBCL], parental mental health [BDI], family structural characteristics [family size, parental education], family relational characteristics [FAD, DAS-32]), *predisposing characteristics* (demographic characteristics [child age, child gender, ethnic background, participants age], religiosity, parental explanations for child problems [PCS], childrearing self-efficacy [PSOC-efficacy]), and *barriers/facilitators* (perceived social support [ISEL], self-reported family income). Univariate analyses examining differences in individual characteristics across levels of help-seeking was conducted using a series of one-way analysis of variance (ANOVA). Analyses were separate for internalizing and externalizing problems, and for mothers and fathers, at the α = 0.05.

A series of multinomial logistic regression models were used to test hypotheses evaluating the individual influence of illness profile, predisposing characteristics, and help-seeking barriers/facilitators on parental help-seeking behaviors for child mental health. Regression modelling was limited to parental concerns with externalizing behavior since fewer parents reported concerns with child internalizing behavior (mother: n = 97; father: n = 69) leading to considerations of inadequate power to detect significant effects. For the analysis, PHSQ responses were recoded into levels of parental help-seeking representing the theoretical stages of help-seeking pathways: response *a—b* → Level 0 ‘no help-seeking from others’, responses *c* → Level 1 ‘family/friends’, responses *d—e* → Level 2 ‘community help-seeking’, and response *f* → Level 3 ‘professional help’ [11, 14].

The first set of models were designed to individually evaluate the determinants of each level of parental help-seeking for externalizing behavior. No help-seeking from others was the reference category. The second set of models were aimed at evaluating determinants associated with parents informal versus professional help-seeking for externalizing behavior. Professional help was the reference category. Independent variables entered into both sets of models were representative of illness profile, predisposing characteristics, and barriers/facilitators, as above. Individual influence of independent variables was assessed by the significance (α = 0.05). Separate analyses were conducted for mothers and fathers.

## Results

### Preliminary Analysis of Parental Help-Seeking for Child Mental Health

#### Distribution of Parental Help-Seeking

Frequencies for various PHSQ response categories for externalizing and internalizing concerns are reported in Table 1 in supplementary information. A summary of levels of parental help-seeking for externalizing and internalizing behaviors are reported in Table 3. For externalizing behaviors, the results indicate that the most common level of parental help-seeking was seeking help from family/friends (mothers: 41.9%; fathers: 37.6%). The next most common level of help-seeking was seeking help from teachers and professional help (mothers: teachers—23.1%, professional help—24.1%; fathers: teachers—18.6%, professional help—14.8%). It is noted that fathers uniquely indicated ‘no help sought’ (17.9%) in almost equal proportion to seeking help from teachers and professionals. Seeking help via methods of self-help or community leaders were the least reported. For internalizing behaviors, levels of parental help-seeking were different for mothers and fathers. Mothers’ responses indicated that the most common level of help-seeking was professionals (44.3%), followed by family/friends (24.7%) and teachers (20.6%). Fathers’ responses indicated that the most common level of help-seeking was family/friends (40.6%), followed by teachers (21.7%) and professionals (17.4%). The least common responses for both mothers and fathers were no help sought, self-help, and community leaders.

**Table 3:** Descriptive statistics of parental concerns for externalizing and internalizing behavior and related help-seeking behaviors

Help-seeking	Externalizing behavior (%)	Internalizing behavior (%)
Mothers		
No concern	91 (23.1)	297 (75.4)
Help sought for concern		
No help sought	16 (5.3)	6 (6.2)
Self-help	10 (3.3)	1 (1)
Family/friends	127 (41.9)	24 (24.7)
Teacher	70 (23.1)	20 (20.6)
Community leader	7 (2.3)	3 (3.1)
Professional help	73 (24.1)	43 (44.3)
Total	303 (76.9)	97 (24.6)
Fathers		
No concern	131 (33.2)	325 (82.5)
Help sought for concern		
No help sought	47 (17.9)	7 (10.1)
Self-help	23 (8.7)	7 (10.1)
Family/friends	99 (37.6)	28 (40.6)
Teacher	49 (18.6)	15 (21.7)
Community leader	6 (2.3)	0 (0)
Professional help	39 (14.8)	12 (17.4)
Total	263 (66.8)	69 (17.5)

##### Univariate Analysis of Parental Help-Seeking

Results from univariate analysis are summarized in Tables 2 and 3 in supplementary information. Of the variables included in the analysis for mothers’ concerns for externalizing behavior, significant differences between help-seeking levels were identified for externalizing symptoms, child age, child responsible attributions, and self-efficacy for childrearing. Mothers who sought professional help reported higher average externalizing problems, *M* = 54.56 (8.16), compared to mothers who did not seek help from others, *M* = 48.00 (7.58), and mothers who sought help from family/friends, *M* = 49.88 (8.73). Mothers who sought professional help also had younger children, *M* = 5.06 (1.42), than those who sought help in the community, *M* = 5.85 (1.49). Mothers who sought help from family/friends, *M* = 2.92 (0.91), in the community, *M* = 3.12 (0.92), and professional help, *M* = 3.29 (1.02), had higher scores for child-responsible attributions compared to mothers who sought no help from others, *M* = 2.31 (0.93). Finally, mothers who sought professional help, *M* = 4.01 (0.98), had lower self-efficacy for childrearing compared to mothers who did not seek help from others, *M* = 4.69 (0.94). Although omnibus tests indicated significant differences across help-seeking levels for child internalizing problems, comparisons were not significant according to the Scheffe method. Furthermore, there were no significant differences between help-seeking levels in variables in the analyses for mothers’ concerns for internalizing behavior.

No significant differences between levels of fathers’ help-seeking for concerns with externalizing behaviors were found in relation to variables representing illness profile, predisposing factors, and barriers/facilitators. Although omnibus tests indicated significant differences across help-seeking levels for child externalizing problems and child gender, comparisons were not significant according to the Scheffe method and Bonferroni correction, respectively. There were no significant differences between help-seeking levels in variables included in the analysis for fathers’ concerns with child internalizing behaviors.

### Multinomial Regression Modelling examining Determinants of Parental Help-Seeking

#### Mothers’ Help-Seeking

Results of multinomial logistic regression examining determinants of each level of mothers’ help-seeking for child externalizing behaviors (reference category was no help-seeking from others) are presented in Table 4. The fit of the final model was significant, explaining 31% of the variance in help-seeking outcomes, χ^2^(51) = 101.45, Nagelkerke R^2^ = 0.31. Mothers who reported less satisfying marriages, OR = 0.96, 95% CI [0.92,1.00], and lower family income, OR = 0.26, 95% CI [0.08,0.88], were more likely to seek help from family/friends compared to no help-seeking from others. Mothers who reported fewer internalizing problems, OR = 0.91, 95% CI [0.85,0.99], less satisfying marriages, OR = 0.95, 95% CI [0.91,0.99], and more years of education, OR = 1.31, 95% CI [1.01,1.70], were more likely seek help in the community compared to no help-seeking. Mothers who reported greater child responsible attributions were more likely to seek professional help compared to no help-seeking, OR = 2.41, 95% CI [1.13,5.17].

**Table 4:** Results of multinomial logistic regression examining determinants of mothers’ level of help-seeking for externalizing concern

	Family/Friends vs. No help-seeking^c^		Community vs. No help-seeking^c^		Professional help vs. No help-seeking^c^	
	B	OR [95% CI]	B	OR [95% CI]	B	OR [95% CI]
Illness profile						
CBCL externalizing symptoms	− 0.01	1.00 [0.92, 1.07]	0.05	1.06 [0.98, 1.14]	0.05	1.05 [0.97, 1.14]
CBCL internalizing symptoms	− 0.06	0.95 [0.88, 1.01]	− 0.09*	0.92 [0.85, 0.99]	− 0.03	0.97 [0.90, 1.05]
Family size	− 0.23	0.80 [0.54, 1.17]	− 0.35	0.71 [0.46, 1.08]	− 0.24	0.79 [0.51, 1.20]
Parental education	0.08	1.08 0[.84, 1.38]	0.27*	1.31 [1.01, 1.70]	0.12	1.12 [0.87, 1.46]
Marital quality	− 0.05*	0.96 [0.92, 1.00]	− 0.05*	0.95 [0.91, 0.99]	− 0.04	0.96 [0.92, 1.00]
Family adjustment	− 1.03	0.36 [.08,1.58]	− 1.02	0.36 [0.08, 1.69]	− 1.15	0.32 [0.07, 1.54]
BDI	0.01	1.01 [0.93, 1.09]	0.01	1.01 [0.92, 1.10]	0.02	1.02 [.94, 1.11]
Predisposing factors						
Child age	0.00	1.00 [0.71, 1.40]	0.34	1.40 [0.97, 2.03]	− 0.13	0.87 [ .60, 1.27]
Child gender: Male^a^	− 0.38	0.68 [0.26, 1.77]	− 0.24	0.79 [0.29, 2.16]	− 0.18	0.83 [0.30, 2.30]
Parents age	0.04	1.04 [0.92, 1.17]	0.03	1.03 [0.91, 1.17]	0.05	1.05 [0.93, 1.20]
Ethnicity: White^b^	0.70	2.01 0[.61, 6.65]	0.25	1.29 [0.37, 4.52]	0.58	1.79 [.49,6.48]
Religiosity	− 0.25	0.78 [0.55, 1.11]	− 0.21	0.81 [0.56, 1.17]	− 0.13	0.88 [0.60, 1.28]
Child responsible attributions	0.50	1.64 [0.79, 3.40]	0.76	2.13 [1.00, 4.55]	0.88*	2.41 [1.13, 5.17]
Parent causal attributions	0.66	1.93 [0.78, 4.77]	0.30	1.35 [0.52, 3.50]	− 0.30	0.74 [0.29, 1.92]
General self-efficacy	− 0.31	0.73 [.038,1.40]	− 0.39	0.68 [0.34, 1.35]	− 0.65	0.52 [0.27,1.03]
Barriers/facilitators						
Family income	− 1.35*	0.26 [0.08,.88]	− 1.13	0.32 [0.09, 1.17]	− 0.76	0.47 [0.13, 1.73]
Social support	0.09	1.09 [0.99, 1.20]	0.09	1.1 [0.99, 1.22]	0.10	1.11 [0.99, 1.24]

Community help-seeking consists of help-seeking from teachers of community leaders

*CBCL* Child Behavior Checklist

*p-value < .05

^a^reference category for gender is 2 = female

^b^reference category for gender is 2 = minority; ^c^Reference category for multinomial regression analysis is no help-seeking (none or self-help)

Results of multinomial logistic regression examining determinants of mothers’ informal versus professional help-seeking for child externalizing behaviors (reference category was professional help) are presented in Table 5. The fit of the final model was significant, explaining 23% of the variance in help-seeking, χ^2^(34) = 62.57, Nagelkerke R^2^ = 0.23. Mothers who reported fewer externalizing problems, OR = 0.95, 95% CI [0.90,0.99], and more parent-causal attributions, OR = 2.65, 95% CI [1.48,4.76], were more likely to seek help from family/friends compared to professional help. Further, mothers who reported fewer internalizing problems, OR = 0.94, 95% CI [0.90,0.99], or an older child, OR = 1.61, 95% CI [1.22,2.12], were more likely to seek help in the community compared to professional help.

**Table 5:** Results of multinomial logistic regression examining determinants of mothers’ non-professional versus professional help-seeking for externalizing concerns

	Family/Friends vs. Professional help^c^		Community vs. Professional help^c^	
	B	OR [95% CI]	B	OR [95% CI]
Illness profile				
CBCL externalizing symptoms	− 0.06*	0.95 [.90,.99]	0.00	1.00 [0.95, 1.06]
CBCL internalizing symptoms	− 0.03	0.97 [0.93,1.02]	− 0.06*	0.94 [0.90, 0.99]
Family size	− 0.02	0.99 [0.73, 1.33]	− 0.13	0.88 [0.63, 1.23]
Parental education	− 0.03	0.97 [0.83, 1.14]	0.16	1.17 [0.99, 1.40]
Marital quality	− 0.01	1.00 [0.97, 1.02]	− 0.01	0.99 [0.97, 1.02]
Family adjustment	0.13	1.14 [0.45, 2.94]	0.12	1.13 [0.41, 3.11]
Depression	− 0.01	0.99 [0.94, 1.04]	− 0.01	0.99 [0.94, 1.04]
Predisposing factors				
Child age	0.14	1.15 [0.90, 1.48]	0.47*	1.61 [1.22, 2.12]
Child gender: Male^a^	− 0.24	0.78 [0.41, 1.49]	− 0.09	0.92 [0.45, 1.85]
Parents age	− 0.02	0.98 [0.91, 1.06]	− 0.03	0.97 [0.89, 1.06]
Ethnicity: White^b^	0.11	1.12 [0.46, 2.74]	− 0.29	0.75 [0.29, 1.93]
Religiosity	− 0.12	0.89 [0.70, 1.13]	− 0.08	0.92 [0.71, 1.19]
Child responsible attributions	− 0.39	0.68 [0.43, 1.06]	− 0.13	0.88 [0.54, 1.41]
Parent causal attributions	0.98*	2.65 [1.48, 4.76]	0.62	1.86 [0.99, 3.50]
General self-efficacy	0.35	1.42 [0.97, 2.08]	0.27	1.31 [0.85, 2.02]
Barriers/facilitators				
Family income	− 0.53	0.59 [0.27, 1.29]	− 0.33	0.72 [0.31, 1.68]
Social support	− 0.02	0.98 [0.91, 1.06]	− 0.01	0.99 [0.91, 1.07]

Community help-seeking consists of help-seeking from teachers of community leaders

*CBCL* Child Behavior Checklist

^*^p-value < .05

^a^Reference category for gender is 2 = female

^b^reference category for gender is 2 = minority; ^c^Reference category for multinomial regression analysis is professional help

#### Fathers’ Help-Seeking

Results of multinomial logistic regression examining determinants of each level of fathers’ help-seeking for child externalizing behaviors (reference category was no help-seeking) are presented in Table 6. The fit of the final model was nonsignificant, explaining 22% of the variance in help-seeking, χ^2^(51) = 59.03, Nagelkerke R^2^ = 0.22. Despite this, the results indicate that fathers of a younger child, OR = 0.76, 95% CI [0.59,0.97] were more likely to seek help from family/friends compared to no help-seeking from others. Fathers who reported more externalizing problems, OR = 1.11, 95% CI [1.04,1.18], and a male child, OR = 3.31, 95% CI [1.46,7.52], were more likely seek help in the community compared to no help-seeking from others. Finally, fathers of a male child were more likely to seek professional help compared to no help-seeking from others, OR = 2.53, 95% CI [1.05,6.12].

**Table 6:** Results of multinomial logistic regression examining determinants of fathers’ level of help-seeking for externalizing concern

	Family/Friends vs. No help-seeking^c^		Community vs. No help-seeking^c^		Professional help vs. No help-seeking^c^	
	B	OR [95% CI]	B	OR [95% CI]	B	OR [95% CI]
Illness profile						
Externalizing symptoms	0.05	1.06 [1.00,1.12]	0.10*	1.11 [1.04,1.18]	0.02	1.02 [0.95,1.10]
Internalizing symptoms	− 0.02	0.98 [.94,1.03]	− 0.04	0.96 [0.91,1.02]	− 0.01	0.99 [0.93,1.05]
Family size	0.21	1.23 [0.91,1.67]	0.02	1.02 [0.71,1.46]	0.06	1.06 [0.71,1.60]
Parental education	− 0.04	0.96 [0.83,1.12]	− 0.03	0.98 [0.81,1.18]	− 0.10	0.91 [0.74,1.11]
Marital quality	− 0.01	0.99 [0.96,1.02]	− 0.01	0.99 [0.96,1.03]	− 0.01	0.99 [0.96,1.03]
Family adjustment	− 0.07	0.93 [0.35,2.51]	− 0.48	0.62 [0.19,2.02]	0.39	1.48 [0.41,5.28]
Depression	− 0.02	0.98 [0.91,1.05]	0.00	1.00 [0.92,1.08]	0.02	1.02 [0.94,1.12]
Predisposing factors						
Child age	− 0.28*	0.76 [0.59,0.97]	0.00	1.00 [0.74,1.35]	− 0.23	0.79 [0.58,1.09]
Child gender: Male^a^	0.28	1.32 [.66,2.64]	1.20*	3.31 [1.46,7.52]	0.93*	2.53 [1.05,6.12]
Parents age	0.00	1.00 [0.94,1.07]	− 0.04	0.96 [0.89,1.04]	− 0.02	0.98 [0.91,1.07]
Ethnicity: White^b^	0.03	1.03 [0.42,2.54]	− 0.78	0.46 [0.17,1.22]	0.54	1.72 [0.46,6.39]
Religiosity	0.08	1.09 [0.88,1.34]	0.19	1.20 [0.93,1.56]	0.16	1.18 [0.89,1.56]
Child responsible attributions	− 0.07	0.93 [0.54,1.61]	− 0.18	0.84 [0.44,1.60]	0.27	1.32 [0.67,2.59]
Parent causal attributions	0.42	1.53 [0.81,2.88]	0.04	1.04 [0.50,2.18]	0.03	1.03 [0.46,2.31]
General self-efficacy	0.15	1.17 [0.79,1.72]	− 0.27	0.76 [0.49,1.20]	0.26	1.3 [0.76,2.20]
Barriers/facilitators						
Family income	− 0.24	0.79 [0.38,1.64]	− 0.50	0.61 [0.26,1.42]	− 0.27	0.77 [0.29,1.99]
Social support	0.04	1.04 [0.96,1.13]	0.04	1.04 [0.95,1.15]	0.03	1.03 [0.93,1.14]

Community help-seeking consists of help-seeking from teachers of community leaders

^*^p-value < .05

^a^reference category for gender is 2 = female

^b^reference category for gender is 2 = minority; ^c^Reference category for multinomial regression analysis is no help-seeking (none or self-help)

Results of multinomial logistic regression examining determinants of informal versus professional help-seeking for fathers’ concerns with child externalizing behaviors (reference category was professional help) are presented in Table 7. The fit of the final model was nonsignificant, explaining 20% of the variance in help-seeking, χ^2^(34) = 37.23, Nagelkerke R^2^ = 0.20. Furthermore, no variables representing illness profile, predisposing factors, or barriers/facilitators were significantly associated with the likelihood of seeking help from either family/friends or in the community compared to professional help.

**Table 7:** Results of multinomial logistic regression examining determinants of fathers’ non-professional versus professional help-seeking for externalizing concerns

	Family/Friends vs. Professional help^c^		Community vs. Professional help^c^	
	B	OR [95% CI]	B	OR [95% CI]
Illness profile				
Externalizing symptoms	0.03	1.03 [0.96, 1.10]	0.08	1.08 [1.00, 1.17]
Internalizing symptoms	− 0.01	0.99 [0.93, 1.05]	− 0.04	0.96 [0.90, 1.03]
Family size	0.17	1.19 [0.81, 1.74]	− 0.10	0.91 [0.59, 1.40]
Parental education	0.03	1.03 [0.85, 1.26]	0.08	1.08 [0.86, 1.36]
Marital quality	0.00	1.00 [0.96, 1.03]	0.00	1.00 [0.96, 1.04]
Family adjustment	− 0.47	0.63 [0.18, 2.18]	− 0.83	0.44 [0.11, 1.78]
Depression	− 0.04	0.96 [0.88, 1.05]	− 0.02	0.98 [0.89, 1.09]
Predisposing factors				
Child age	− 0.06	0.94 [0.69, 1.27]	0.26	1.30 [0.92, 1.84]
Child gender: Male^a^	− 0.66	0.52 [0.23, 1.18]	0.34	1.40 [0.54, 3.62]
Parents age	0.02	1.02 [0.95, 1.11]	− 0.03	0.97 [0.89, 1.06]
Ethnicity: White^b^	− 0.41	0.67 [0.19, 2.37]	− 1.27	0.28 [0.08, 1.05]
Religiosity	− 0.10	0.91 [0.70, 1.19]	− 0.03	0.98 [0.72, 1.33]
Child responsible attributions	− 0.44	0.65 [0.33, 1.27]	− 0.40	0.67 [0.31, 1.47]
Parent causal attributions	0.47	1.60 [0.74, 3.45]	− 0.09	0.92 [0.38, 2.19]
General self-efficacy	− 0.06	0.94 [0.56, 1.56]	− 0.55	0.58 [0.33, 1.02]
Barriers/facilitators				
Family income	0.00	1.00 [0.39, 2.53]	− 0.23	0.79 [0.29, 2.16]
Social support	0.01	1.01 [0.92, 1.11]	0.00	1.00 [0.9, 1.12]

Community help-seeking consists of help-seeking from teachers of community leaders

*p-value < .05

^a^reference category for gender is 2 = female

^b^reference category for gender is 2 = minority

^c^Reference category for multinomial regression analysis is professional help

## Discussion

The aim of the current study was to examine parents’ help-seeking for concerns with child externalizing and internalizing behaviors in the community. Parents reported having substantially more concerns with child externalizing compared to internalizing behaviors. For externalizing behaviors, both mothers and fathers were most likely to seek help from family/friends, followed by teachers and professionals. For internalizing behaviors, mothers were most likely to seek help from professionals followed by family/friends and teachers, while fathers were most likely to seek help from family/friends followed by teachers and professionals. Mothers’ informal help-seeking was associated with specific variables representing illness profile (i.e., marital quality, child mental health, parental education), predisposing factors (parent-causal attributions, child age), and family income, while professional help-seeking was associated child-responsible attributions. Parent and family functioning seem to hold little value in modelling mothers’ help-seeking. Fathers informal and professional help-seeking was associated with child demographics (age, gender), with little information available to identify determinants of informal versus professional help-seeking.

The results converge with previous research suggesting that parents prefer to seek help from an informal sphere that predominantly includes family/friends compared to seeking help from professionals for child mental health concerns [5, 14, 18, 19]. However, the current results also uniquely identified that parents’ help-seeking preferences may be different for mothers and fathers. For instance, although both mothers and fathers reported similar rates of help-seeking from family/friends for child externalizing behaviors, fathers also reported a preference to not seek help from others at all, which was not the same as mothers. Furthermore, mothers reported seeking professional help for child internalizing behaviors more often than fathers, while fathers were inclined to not seek help for child internalizing behaviors. Taken together, these results suggest that increased recognition of individual differences between mothers’ and fathers’ help-seeking is needed to understand families’ ultimate decisions to seek different care for child mental health problems.

Analysis of the general distribution of parental help-seeking also suggested that if parents do decide to seek help from an authoritative figure, they are likely to seek help from teachers as much as from healthcare professionals. This was especially the case for mothers’ and fathers’ concerns with externalizing behaviors, which is consistent with previous research which has indicated that accessing treatments for behavior problems in nonclinical community settings may help overcome barriers to treatment [20]. This is also consistent with intervention implementation work that seeks to train teachers and others to deliver evidence-based parenting interventions [32, 33]. It could be that by arming teachers to provide optimal advice, we could substantially facilitate parents’ access to effective mental health intervention. This is consistent with suggestions of task shifting whereby the mental health workforces is expanded to include lay individuals to provide support that otherwise might be delivered by health professionals [6].

Importantly, the current findings suggest that child and family differences may set the stage for different levels of parental help-seeking for child mental health. The salience of illness profile was emphasized for externalizing behaviors; namely, lower quality marital relationships, higher parental education, lower internalizing symptoms, alongside lower family income, all being linked with mothers seeking informal help. These results converge with previous literature showing that illness profile is a key predictor of parental help-seeking [16]. However, they contrast findings of parental help-seeking for social-emotional development among young children (3 years old), which emphasized parent demographics and previous service use [18]. This may suggest that mothers’ perception of need and family impact may be especially important to understanding mothers’ decision to seek help for mental health problems that emerge in mid-childhood, especially among families with lower incomes. In relation to seeking professional help, the influence of child and family characteristics seems to shift from subjective assessments of child problems to mothers’ attributions for child problems, which converges with recent findings suggesting that mothers’ explanations for child behavior is influential in professional help-seeking [34, 35]. Mothers who view the child’s behaviors as things the child does intentionally and are under her/his control appears to be a catalyst for seeking help from professionals [36]. For fathers, we note a general emphasis on child demographics of age and gender in influencing informal and professional help-seeking. These findings for fathers are novel, as specific findings for fathers have never been previously reported. The results reinforce the importance of considering differences between mothers and fathers in studying parental help-seeking for child mental health.

### Implications for Theory, Policy, and Clinical Practice

Overall, the current findings reinforce and help refine models of parental help-seeking specifically for child mental health by highlighting that it is the *mothers’* subjective assessment of clinical problems and attributions for their children’s problems which is associated with whether help is sought, and the type of help sought. These findings are generally consistent with previous models [17] which have suggested that the more complex and severe the children’s mental health problem is, the more likely families seek help. However, the specific role of mothers’ attributions was highlighted as a salient predictor of help-seeking levels, with the current findings suggesting that the extent to which mothers attribute child problems to either the child or themselves significantly relates to the type of help sought for child mental health problems. Mothers who seek professional help may be more likely to believe externalizing problems is caused by child factors, which could be a key factor in the types of supports requested [37]. For instance, child-blaming attributions for problems may reduce mother’s willingness to participate in parent-directed treatment (e.g., behavioural parent training) that are well-established for externalizing problems [36]. As such, practitioners should assess and monitor parental attributions at the point at which professional help is sought by mothers to help manage their expectations for treatment [37].

It is important to note that none of the variables measuring illness profile, predisposing characteristics, and barriers/faciilitators were significantly linked with fathers’ help-seeking, raising questions of the relevance of these theoretical-based variables in influencing fathers’ help-seeking behaviors. It could be that mothers (a) absorb more of the family impact of child problems, (b) take the lead in deciding what triggers help-seeking and the type of help needed for children, or (c) both. Research into parental roles in families suggest that while fathers are more involved in parenting than ever before, the mother’s role entails crisis manager during times of family distress, including seeking professional help [38, 39]. To that end, it may be that fathers are more likely to “go along” with mothers’ decisions and thus, relations with predictors are more apparent for mothers as compared with fathers [20].

There are several other salient implications for theory from these findings in understanding parental help-seeking processes that may help address the treatment gap in child mental health. For instance, the results highlight models of parental help-seeking for child mental health should acknowledge that a great deal of parental help-seeking is informal. This is potentially important to improving access to mental health support, since seeking informal help from family/friends and teachers may represent an avenue to interact with evidence-based advice and open crucial referral pathways to seeking higher levels of support from professionals [11, 14, 17]. Informal sources of help then potentially become a facilitator of appropriate help-seeking rather than a reason for the treatment gap, if individuals in the community are equipped with appropriate literacy of child mental health symptoms and evidence-based treatment options [40], or even have the ability to deliver “bite-sized” doses of evidence-based treatment. Furthermore, there is an interesting range in the child and family factors that relate to mothers’ decisions to seek different levels of help for child mental health concerns. There is need therefore for further consideration of putative moderating factors to help match treatment delivery (i.e., setting, locations, formats) to individual characteristics. For instance, the developmental stage of the child may influence which child and family characteristics predict decision to seek help. Findings that parent and family functioning did not discriminate between different types of help-seeking diverges from expectations that greater maternal and family distress is usually associated with professional help [16]. We posit that the generally younger age range in the current sample may constrain the full range of impacts that families with older children might experience.

### Study Limitations and Future Directions

Despite the significant findings of this study, it is not without limitations. Our data are all from a single time point and our analyses are cross-sectional and correlational. As such, we cannot speak to causality or temporal sequence. Our sample was a generally representative community sample of families in a suburban area. This allows us to examine the relation between symptom severity and help-seeking using the entire range of symptoms. However, it does, inherently limit our power to study these relations because the majority of children do not evidence mental health problems. Thus, despite having nearly 400 families included in our analyses; we were underpowered to test for differences in help-seeking for internalizing problems. Also, because this was a community sample, we do not have independent clinical evaluations of child functioning and are limited to mothers’ and fathers’ reports. Importantly, we focused on seeking help from others. We cannot address help-seeking from informational sources such as the internet and books. Finally, our results are not generalizable to populations that may be of special interest and cannot be isolated within our sample with enough power to analyze separately, such as families living in poverty. Families with single parents or same-sex parenting structures were specifically excluded from the study and, while this study uniquely investigated inter-parental differences between mothers and fathers, we did not collect information regarding gender identity (e.g., transgender, gender nonconforming) and therefore the results cannot be generalized to these populations as well. Despite these limitations, however, we believe the strengths of this study support its contribution to the literature. This is the first study to examine both mothers’ and fathers’ help-seeking behaviors and predictors of these behaviors. This helped highlight inter-parental differences in processes of parental help-seeking. Moreover, the current study used gold-standard random sampling procedures in the community to optimize representativeness of these families in the population.

Suggestions for future research entail leveraging longitudinal data to address the aforementioned limitations of the current study. For instance, longitudinal data will be helpful in isolating the direction of effects over time. This research will be enhanced by stratified sampling methodology to ensure sufficient representation of diverse groups, such as children with clinically-elevated mental health problems and socially diverse families (non-urban, single-sex couples, single parents, low socioeconomic status, transgender, gender nonconforming). We also recommend using well-established population datasets, such as the Millennium Cohort Study or Avon Longitudinal Study of Parents and Children in the United Kingdom as examples, whereby there would be sufficient power to investigate determinants of parental help-seeking in special interest groups. Data linkage with electronic educational and health records could then be used to examine determinants of actual help-seeking behavior, which would provide a more reliable analysis of models of parental help-seeking. Finally, future research should continue to investigate the salience of inter-parental differences in help-seeking for child problems, including how much mother’s versus father’s preferences weigh in to whether help was sought.

## Summary

The current study examined mothers’ and fathers’ help-seeking for child externalizing and internalizing behavior to help extend research addressing the treatment gap in child mental health. Results indicated that a large proportion of parents prefer seeking informal help from family and friends, especially for child externalizing problems. Further, mothers’ rather than fathers’ subjective assessment of clinical problems and attributions for their child’s problems was important to whether help was sought, and the type of help sought. Based on the current results, we propose that sources of informal help may potentially facilitate rather than hinder appropriate help-seeking for child mental health, if individuals in the community are equipped with appropriate literacy of child mental health symptoms (especially improved understanding of causes of behavior) and evidence-based treatment options [40], or even have the ability to deliver “bite-sized” doses of evidence-based treatment (i.e., task shifting). Furthermore, it is important to consider potential moderating factors such as developmental stage of the child, as well as the interactive influence of fathers, in matching families to different levels of help. To that end, investment into researching appropriate task-shifting and matching of families to levels of mental health support may represent a promising avenue for reducing the treatment gap.

## Supplementary Information

Below is the link to the electronic supplementary material.

Supplementary file1 (DOCX 42 KB)

## Data Availability

Data are not available for 3rd party use or distribution.
