# Supplementary material for: The Ws of Parental Help-Seeking: When, Where, and for What Do Parents Seek Help for Child Mental Health
Source: Child Psychiatry Hum Dev. 2024 Mar 20;57(1):25–38. doi: 10.1007/s10578-024-01683-5 (PMC12971890; doi:10.1007/s10578-024-01683-5)
Supplement: Supplementary file 1 — Supplementary file1 (DOCX 42 KB) [file 10578_2024_1683_MOESM1_ESM.docx]

## CHS

**Parental Help-Seeking Questionnaire (PHQ)**

In this next section, what we would like you to do is think of all the times you had been concerned about something about **your target child in the past year**. This could be something minor such as your child misbehaved or this could be something larger such as your child throwing tantrums. So, what we would like you to do is list the five things that come to mind (or as many as you can think of) that were of concern to you about **your target child in the past year**. **After you list each one, please check with whom you talked to about this concern**.

Concerns or problems with my **target child in the past year**:

Concern1.__________________________________________________________________________________________________________________________________________________________

Which of the following did you do: (please check all that apply)

I sought no help, (I didn’t talk to anyone, and I didn’t look for any information).

I tried to work it out by myself by reading books, searching on in the

internet, or self help methods.

I talked to friends and family about it.

I talked to my child’s teacher.

I talked to someone from the community, such as a minister or group

leader. What sort of person?_____________________________________

I talked to a professional, such as a therapist or pediatrician.

What sort of professional?___________________________________________

Concern2._________________________________________________________________________________________________________________________________________________________

Which of the following did you do: (please check all that apply)

I sought no help, (I didn’t talk to anyone, and I didn’t look for any information).

I tried to work it out by myself by reading books, searching on in the

internet, or self help methods.

I talked to friends and family about it.

I talked to my child’s teacher.

I talked to someone from the community, such as a minister or group

leader. What sort of person?_____________________________________

I talked to a professional, such as a therapist or pediatrician.

What sort of professional?___________________________________________

Concern3.__________________________________________________________________________________________________________________________________________________________

Which of the following did you do: (please check all that apply)

I sought no help, (I didn’t talk to anyone, and I didn’t look for any information).

I tried to work it out by myself by reading books, searching on in the

internet, or self help methods.

I talked to friends and family about it.

I talked to my child’s teacher.

I talked to someone from the community, such as a minister or group

leader. What sort of person?_____________________________________

I talked to a professional, such as a therapist or pediatrician.

What sort of professional?___________________________________________

Concern4.___________________________________________________________________________________________________________________________________________________________

Which of the following did you do: (please check all that apply)

I sought no help, (I didn’t talk to anyone, and I didn’t look for any information).

I tried to work it out by myself by reading books, searching on in the

internet, or self help methods.

I talked to friends and family about it.

I talked to my child’s teacher.

I talked to someone from the community, such as a minister or group

leader. What sort of person?_____________________________________

I talked to a professional, such as a therapist or pediatrician.

What sort of professional?___________________________________________

Concern5.___________________________________________________________________________________________________________________________________________________________

Which of the following did you do: (please check all that apply)

I sought no help, (I didn’t talk to anyone, and I didn’t look for any information).

I tried to work it out by myself by reading books, searching on in the

internet, or self help methods.

I talked to friends and family about it.

I talked to my child’s teacher.

I talked to someone from the community, such as a minister or group

leader. What sort of person?_____________________________________

I talked to a professional, such as a therapist or pediatrician.

What sort of professional?___________________________________________

**Protocol for coding parental concerns**

**Externalizing (e)** is acting out - oppositional, disruptive, hyper, aggressive behavior as well as inattention

**Differentials:**

- not 'he teases his sister" but include "he fights with his sister" and "he steals her toys"
- Include "can't follow directions" because it sounds oppositional-ish
- Responses that sound as though they are in opposition to parents’ wishes
  - E.g., "cleaning his room", "running away at the store, getting lost", "looking both ways when riding or playing in the street", "not eating properly at right time or enough", "child is too dependent on adults, including cleaning up mess".
- Include eating items that sound oppositional
  - E.g., eats too much candy, won't try foods, things like that
- Don’t include eating items that are indicators of picky eating like "won't eat vegetables” or vague, like “eating habits”
- For sleep issues, include items that have an oppositional feel like “bedtime” and “won’t go to bed”, “likes to sleep in our bed”
  - Don’t include items that indicate general sleep issues such as “difficulty sleeping”, and “doesn’t get enough sleep” unless they are written to indicate e or i

**Internalizing (i)** is sad or anxious (for it to count it needs to either have a sad or anxious flavor or suggest a change - a pulling back)

-Somatic complaints such as stomach aches, frequent trips to the school nurse, “pain in chest”

**Differentials:**

- Include withdrawn, but not just quiet or shy.
- Include nail-biting but not thumb sucking
- Do not include "she gets picked on" here UNLESS it's that she gets upset by how other kids treat her
- Bathroom-related concerns should be other unless there is a clear indicator of anxious flavor to it
  - E.g., “won't use a public bathroom”, “can't pee while at school”
- Somatic items that give no indications of it being internalizing (e.g., "my son's bedwetting" and "potty training”) should be other
- Somatic items that could be due to e or i such as "bowel control, over control, doesn't like to go" and "urination in classroom (wetting self in school) should be other

**School (s)** is cognitive or academic - speech issues, slow to learn to read, school problems that are not e or i that include something that could impact academics. doesn't listen to teacher, etc. doesn't keep homework organized.

- Vague references to school or academic topics

**Sleep (sleep)** is any issue related to sleep difficulties

Code = as e, i, or o and then 1 in next column

**Other (o)** is any response that is not e, i, s

Also utilize when the response is not clear whether it would be e or i but it is likely one of them

Supplementary information Table 1

*Frequencies for various PHSQ response categories for parental concerns of externalising and internalising behaviour.*

| Help-seeking | Externalizing behavior | Internalizing behavior |
| --- | --- | --- |
| *Mothers* |  |  |
| No help sought | 57 (8.8%) | 10 (4.9%) |
| Self-help | 147 (22.6%) | 32 (15.8%) |
| Family/friends | 253 (38.9%) | 75 (36.9%) |
| Teacher | 110 (16.9%) | 38 (18.7%) |
| Community leader | 11 (1.7%) | 5 (2.5%) |
| Professional help | 73 (11.2%) | 43 (21.2%) |
| *Fathers* |  |  |
| No help sought | 90 (18.8%) | 9 (8.5%) |
| Self-help | 102 (21.3%) | 23 (27.7%) |
| Family/friends | 168 (35.1%) | 43 (40.6%) |
| Teacher | 72 (15.1%) | 18 (17.0%) |
| Community leader | 7 (1.5%) | 1 (0.9%) |
| Professional help | 39 (8.2%) | 12 (11.3%) |

Supplementary information Table 2

|  | **Mothers helpseeking for**  **externalising behaviour** | | | | | **Mothers helpseeking for**  **internalising behaviour** | | | | |
| --- | --- | --- | --- | --- | --- | --- | --- | --- | --- | --- |
| **Level of helpseeking** | **0** | **1** | **2** | **3** | **Statistic^a^** | **0** | **1** | **2** | **3** | **Statistic^b^** |
| *Illness profile* |  |  |  |  |  |  |  |  |  |  |
| Externalising CBCL | 48.00 (7.58)^†^ | 49.88 (8.73)^‡^ | 53.29 (9.77) | 54.56 (8.16)^†‡^ | 6.76* | 50.71 (10) | 48.00 (8.42) | 49.22 (7.99) | 50.81 (9.42) | .57 |
| Internalising CBCL | 48.50 (9.40) | 48.06 (9.17) | 49.01 (8.77) | 51.81 (9.77) | 2.65* | 51.14 (6.15) | 52.46 (9.40) | 51.3 (7.62) | 51.12 (9.33) | .13 |
| Family size | 5.19 (1.63) | 4.66 (1.24) | 4.52 (1.19) | 4.48 (.96) | 2.51 | 5.00 (1.16) | 4.50 (.72) | 4.65 (1.4) | 4.30 (.96) | 1.19 |
| Parental education | 13.85 (1.52) | 13.97 (2.23) | 14.61 (2.07) | 14.29 (2.42) | 1.65 | 12.71 (1.89) | 14.38 (2.22) | 14.74 (2.42) | 14.30 (2.25) | 1.44 |
| Marital quality | 112.71 (16.72) | 105.45 (18.49) | 104.13 (17.61) | 105.16 (19.56) | 1.47 | 109.57 (14.94) | 107.54 (15.73) | 102.25 (21.02) | 106.67 (18.6) | .48 |
| Family adjustment | 1.56 (.40) | 1.65 (.51) | 1.65 (.48) | 1.64 (.48) | .26 | 1.9 (.46) | 1.7 (.42) | 1.6 (.38) | 1.67 (.42) | 1.02 |
| BDI | 7.77 (6.59) | 9.11 (7.38) | 8.88 (8.63) | 10.71 (9.2) | 1.14 | 9.86 (8.36) | 10.04 (8.17) | 9.52 (6.88) | 9.41 (8.22) | .04 |
| *Predisposing factors* |  |  |  |  |  |  |  |  |  |  |
| Child age | 5.55 (1.35) | 5.33 (1.46) | 5.85 (1.49)† | 5.06 (1.42)† | 4.01* | 6.28 (1.89) | 5.42 (1.42) | 5.94 (1.35) | 5.37 (1.46) | 1.40 |
| Child gender^c^ |  |  |  |  |  |  |  |  |  |  |
| Male | 13 (4.3%) | 56 (18.5%) | 37 (12.2%) | 36 (11.9%) | .71 | 2 (2.06%) | 14 (14.43%) | 9 (9.28%) | 19 (19.59%) | 2.81 |
| Female | 13 (4.3%) | 71 (23.4%) | 40 (13.2%) | 37 (12.2%) |  | 5 (5.15%) | 10 (10.31%) | 14 (14.43%) | 24 (24.74%) |  |
| Parents age | 35.19 (5.17) | 34.76 (4.64) | 34.97 (4.15) | 34.63 (5.56) | .12 | 38.14 (5.76) | 35.42 (4.2) | 35.87 (2.91) | 33.88 (5.89) | 1.99 |
| Ethnicity^c^ |  |  |  |  |  |  |  |  |  |  |
| White | 18 (5.9%) | 108 (35.6%) | 61 (20.1%) | 61 (20.1%) | 4.16 | 4 (4.12%) | 20 (20.62%) | 19 (19.59%) | 35 (36.08%) | 2.63 |
| Minority | 8  (2.6%) | 19 (6.2%) | 16 (5.3%) | 12 (4.00%) |  | 3 (3.09%) | 4 (4.12%) | 4 (4.12%) | 8 (8.25%) |  |
| Religosity | 4.73 (1.40) | 3.99 (1.34) | 4.13 (1.59) | 4.18 (1.49) | 1.91 | 3.86 (1.68) | 4.29 (1.49) | 4.43 (1.34) | 4.30 (1.55) | .27 |
| Child responsible attributions | 2.31 (.93)^†‡⁋^ | 2.92 (.91)^†^ | 3.12 (.92)^‡^ | 3.29 (1.02)^⁋^ | 7.57* | 2.62 (1.35) | 2.79 (.94) | 2.68 (1.01) | 2.87 (1.02) | .24 |
| Parent causal attributions | 1.97 (.72) | 2.39 (.75) | 2.36 (.74) | 2.27 (.67) | 2.63 | 2.23 (.65) | 2.43 (.84) | 2.27 (.69) | 2.38 (.74) | .28 |
| General self-efficacy for childrearing | 4.69 (.94)^†^ | 4.33 (.91) | 4.29 (.84) | 4.01 (.98)^†^ | 4.04* | 4.39 (.86) | 4.24 (.91) | 4.28 (.67) | 4.24 (.96) | .07 |
| *Barriers/facilitators* |  |  |  |  |  |  |  |  |  |  |
| Family income | 85.53 (29.54) | 79.11 (38.85) | 83.76 (57.91) | 84.98 (37.43) | .40 | 84.64 (31.5) | 79.18 (34.51) | 75.81 (21.54) | 80.60 (47.73) | .12 |
| Social support | 34.42 (5.19) | 35.06 (4.65) | 35.12 (4.68) | 35.25 (5.47) | .18 | 33.86 (3.63) | 36.29 (3.2) | 33.78 (5.04) | 34.91 (4.76) | 1.41 |

*Notes*. Statistics reported are means (standard deviation) for continious variables and frequency (proportion) for categorical variables^c^; Levels of help-seeking: 0 = No help/self-help; 1 = family/friends; 2 = community; 3 = professional help; ^a^Statistics test is *F*(3,299) for continuous variables and χ^2^(1) for categorical variables; ^b^Statistics test is *F*(3,93) for continuous variables and χ^2^(1) for categorical variables child gender and ethnicity; **p*-value < .05; ^†‡⁋^Scheffe post-hoc test result indicating significant difference between groups.

Supplementary information Table 3

|  | **Fathers helpseeking for**  **externalising behaviour** | | | | | **Fathers helpseeking for**  **internalising behaviour** | | | | |
| --- | --- | --- | --- | --- | --- | --- | --- | --- | --- | --- |
| **Level of helpseeking** | **0** | **1** | **2** | **3** | **Statistic^a^** | **0** | **1** | **2** | **3** | **Statistic^b^** |
| *Illness profile* |  |  |  |  |  |  |  |  |  |  |
| Externalising CBCL | 49.71 (9.10) | 53.27 (8.44) | 54.18 (9.84) | 52.79 (9.92) | 3.03* | 48.07 (7.25) | 51.36 (9.18) | 52.27 (10.97) | 50.58 (9.51) | .56 |
| Internalising CBCL | 49.29 (10.38) | 50.81 (9.19) | 50.56 (8.04) | 51.33 (10.59) | .51 | 50.86 (10.34) | 51.5 (7.85) | 53.07 (10.48) | 55.25 (11.56) | .57 |
| Family size | 4.51 (1.07) | 4.65 (1.04) | 4.55 (1.40) | 4.33 (1.11) | .72 | 4.21 (.70) | 4.68 (1.06) | 4.20  (.56) | 4.33  (.99) | 1.38 |
| Parental education | 14.69 (2.24) | 14.43 (2.30) | 14.24 (2.46) | 14.15 (2.47) | .58 | 14.14 (2.74) | 14.54 (1.92) | 15.00 (2.48) | 14.17 (2.25) | .44 |
| Marital quality | 107.65 (16.77) | 105.79 (13.99) | 107.67 (16.81) | 104.66 (15.7) | .48 | 104.93 (13.08) | 108.5 (14.59) | 106.86 (15.73) | 111.93 (15.43) | .53 |
| Family adjustment | 1.69 (.41) | 1.76 (.45) | 1.70 (.44) | 1.81 (.53) | .81 | 1.86 (.48) | 1.85  (.35) | 1.62  (.56) | 1.57  (.47) | 1.8 |
| BDI | 6.7 (6.06) | 6.77 (5.25) | 6.9 (5.35) | 7.76 (5.85) | .35 | 6.57 (5.43) | 8.39 (5.53) | 6.79 (6.15) | 7.67 (5.31) | .44 |
| *Predisposing factors* |  |  |  |  |  |  |  |  |  |  |
| Child age | 5.71 (1.44) | 5.22 (1.37) | 5.67 (1.52) | 5.24 (1.49) | 2.37 | 5.84 (1.13) | 5.53 (1.59) | 6.34 (1.39) | 5.70 (1.37) | 1.08 |
| Child gender^c^ |  |  |  |  |  |  |  |  |  |  |
| Male | 27 (10.27%) | 40 (15.21%) | 33 (12.55%) | 23 (8.75%) | 9.70* | 6  (8.7%) | 11 (15.94%) | 7 (10.14%) | 4  (5.8%) | .54 |
| Female | 43 (16.35%) | 59 (22.43%) | 22 (8.37%) | 16 (6.08%) |  | 8 (11.59%) | 17 (24.64%) | 8 (11.59%) | 8 (11.59%) |  |
| Parents age | 37.80 (5.80) | 36.84 (4.98) | 35.76 (6.34) | 36.56 (6.70) | 1.31 | 37.07 (3.71) | 35.75 (5.30) | 38.47 (6.15) | 36.92 (6.69) |  |
| Ethnicity^c^ |  |  |  |  |  |  |  |  |  |  |
| White | 58 (22.05%) | 82 (31.18%) | 39 (14.83%) | 35 (13.31%) | 6.01 | 11 (15.94%) | 24 (34.78%) | 11 (15.94%) | 9 (13.04%) | 1.18 |
| Minority | 12 (4.56%) | 17 (6.46%) | 16 (6.08%) | 4 (1.52%) |  | 3 (4.35%) | 4  (5.8%) | 4  (5.8%) | 3  (4.35%) |  |
| Religosity | 3.44 (1.67) | 3.48 (1.59) | 3.75 (1.44) | 3.59 (1.43) | .46 | 3.71 (1.9) | 3.46 (1.53) | 3.60 (1.50) | 4.33 (1.07) | .92 |
| Child responsible attributions | 2.67 (.87) | 2.93 (.78) | 2.79 (.81) | 2.98 (.98) | 1.71 | 2.43 (.74) | 2.68  (.71) | 2.74  (.81) | 2.35  (.97) | .88 |
| Parent causal attributions | 2.20 (.60) | 2.38 (.74) | 2.19 (.73) | 2.32 (.82) | 1.3 | 2.17 (.60) | 2.49  (.65) | 2.14  (.76) | 1.91  (.71) | 2.37 |
| General self-efficacy for childrearing | 4.05 (.90) | 4.06 (.88) | 4.00 (.97) | 4.16 (.79) | .27 | 4.24 (.77) | 3.80  (.91) | 4.30  (.89) | 4.21  (.69) | 1.68 |
| *Barriers/facilitators* |  |  |  |  |  |  |  |  |  |  |
| Family income | 89.63 (43.49) | 81.81 (35.69) | 84.03 (68.08) | 77.15 (29.58) | .71 | 76.81 (39.09) | 92.75 (54.65) | 82.35 (45.79) | 65.46 (21.12) | 1.11 |
| Social support | 33.64 (4.61) | 34.46 (4.52) | 34.51 (5.06) | 34.00 (5.35) | .52 | 31.14 (6.02) | 34.50 (3.95) | 32.33 (5.21) | 34.00 (4.07) | 1.87 |

*Notes*. Statistics reported are means (standard deviation) for continious variables and frequency (proportion) for categorical variables^c^; Levels of help-seeking: 0 = No help/self-help; 1 = family/friends; 2 = community; 3 = professional help; ^a^Statistics test is *F*(3,258) for continuous variables and χ^2^(1) for categorical variables; ^b^Statistics test is *F*(3,65) for continuous variables and χ^2^(1) for categorical variables child gender and ethnicity; **p*-value < .05; ^†‡⁋^Scheffe post-hoc test result indicating significant difference between groups.
